# Comparative analysis of amino acid sequence level in plant GATA transcription factors

**DOI:** 10.1038/s41598-024-81159-7

**Published:** 2024-11-30

**Authors:** Mangi Kim

**Affiliations:** Department of Biotechnology, Sangmyung University, 03016 Seoul, Republic of Korea

**Keywords:** Genetic variation, Gene regulation, DNA-binding proteins

## Abstract

Transcription factors (TFs) are essential regulators of gene expression, influencing numerous biological processes such as development, growth, and cellular responses in plants. Among these, GATA TFs are distinguished by their highly conserved DNA-binding domain, characterized by a class IV zinc finger motif (CX_2_CX_18-20_CX_2_C). This study investigates the amino acid sequence patterns of 5,335 GATA TFs across 165 plant species sourced from the PlantTFDB database (http://planttfdb.gao-lab.org/), encompassing diverse taxonomic groups. Through comparative sequence analysis, I identify conserved domains and structural features that enhance the understanding functional roles, evolutionary conservation, and lineage-specific adaptations of GATA TFs. These findings provide valuable insights into the diversification and functional specialization of GATA TFs, with implications for improving stress tolerance and adaptability in crops. This study contributes to the broader knowledge of transcriptional regulation in plant biology.

## Introduction

Plants encompass a vast diversity of species that are broadly classified into several groups, including angiosperms (flowering plants) and gymnosperms (non-flowering seed plants), among others^1^. This classification reflects the evolutionary relationships and morphological differences within the plant kingdom. Of these groups, angiosperms represent the largest and most diverse group, with more species identified than in any other groups. This diversity among angiosperms is attributed to their advanced reproductive structures, which have allowed them to adapt and proliferate in a wide range of environments. Consequently, angiosperms play a dominant role in terrestrial ecosystems, contributing significantly to biodiversity and ecological balance^2^.

Plant transcription factors (TFs) are pivotal proteins that regulate gene expression by binding to specific promoter sequences and interacting with other proteins, including RNA polymerase^3^. These interactions form complex regulatory networks essential for diverse biological functions such as development^4^, environmental response^5^, and cell cycle control^6^. The functional specificity of TFs is largely determined by their amino acid sequences, particularly the presence of key domains such as DNA-binding, activation, regulatory, and interaction domains^7^. Consequently, analyzing the amino acid sequence patterns of TFs is crucial for predicting protein function, understanding evolutionary conservation, and elucidating gene expression mechanisms.

In the realm of plant biology, TFs have been classified into 58 distinct types based on sequence similarity and the tertiary structure of their DNA-binding domains, including well-known families like bZIP, bHLH, ARF, and GATA^8^. Among these, GATA TFs are characterized by a unique DNA-binding domain featuring a class IV zinc finger motif (CX_2_CX_18-20_CX_2_C), which is crucial for binding to specific DNA sequences (WGATAR; W = T or A, R = G or A) and regulating gene transcription^9^. The GATA TF family in plants performs vital functions, such as controlling chloroplast development, growth, and division^10^ through the regulation of numerous target genes. For instance, in *Arabidopsis thaliana*, *AtGATA21* and *AtGATA22* regulate the transcription of 1,475 and 638 target genes^11^, respectively, influencing processes like greening, cold tolerance, and flowering time^12^.

Plant GATA TFs are categorized into several domain types, with the most prominent being type IVb (CX_2_CX_18_CX_2_C) and type IVc (CX_2_CX_20_CX_2_C). GATA TFs having type IVb are found in classes A, B, and D, each contributing to distinct biological functions^13^: class A regulates light and brassinosteroid (BR) signaling essential for seedling development^14^, class B contains the HAN and LLM domains linked to growth and floral development^15,16^, and class D generally have an LXXLL motif, which likely mediates interactions with various TFs, suggesting unique regulatory functions^17^. Meanwhile, GATA TFs having type IVc belong to class C, characterized by TIFY and CCT motifs that play key roles in integrating light information and regulating flowering time^18,19^. These domain variations underscore the functional diversity of GATA TFs and their importance in plant adaptation and developmental processes across species.

To gain deeper insights into the functional diversity and evolutionary dynamics of GATA TFs, I analyzed 5,335 GATA TF sequences from 165 plant species, including angiosperms and non-angiosperms, sourced from the PlantTFDB database. This study aims to comprehensively analyze the amino acid sequence patterns of plant GATA TFs, providing detailed characterizations that enhance the understanding of their structural and functional roles. This analysis not only contributes to fundamental plant biology but also has potential applications in biotechnology and agriculture.

## Results and discussions

### Distribution and diversity of plant GATA genes

A total of 4,762 GATA genes, encoding 5,335 GATA TFs, were identified from 165 plant species using the PlantTFDB database (Table 1 and Table S1). The higher count of GATA TFs compared to GATA genes is attributed to alternative splicing observed in 49 of the 165 plant species, indicating functional diversification within individual GATA genes^20^. The 165 plant species were categorized into nine groups: (i) 3,382 GATA genes (3,742 GATA TFs) from 100 eudicot species, (ii) 1,142 (1,290 GATA TFs) from 39 monocot species, (iii) 20 (20 GATA TFs) from the one basal angiosperm species, (iv) 43 (43 GATA TFs) from five gymnosperm species, (v) 6 (10 GATA TFs) from one marchantiophyta species, (vi) 30 (88 GATA TFs) from two bryophyta species, (vii) 8 (8 GATA TFs) from one lycopodiophyta species, (viii) 10 (10 GATA TFs) from one charophyta species, and (ix) 121 (124 GATA TFs) from fifteen chlorophytae species.

**Table 1:** Information of GATA TFs of 165 plant species.

No	Species	Group	Order	# of GATA genes	# of GATA TFs	# of GATA genes having alternative splicing forms	# of GATA TFs having alternative splicing forms	Refs
1	*Daucus carota*	Eudicots	Apiales	29	29	0	0	–
2	*Artemisia annua*	Eudicots	Asterales	15	15	0	0	–
3	*Helianthus annuus*	Eudicots	Asterales	2	2	0	0	–
4	*Lactuca sativa*	Eudicots	Asterales	22	22	0	0	–
5	*Aethionema arabicum*	Eudicots	Brassicales	23	23	0	0	–
6	*Arabidopsis halleri*	Eudicots	Brassicales	22	23	1	2	–
7	*Arabidopsis lyrata*	Eudicots	Brassicales	28	28	0	0	–
8	*Arabidopsis thaliana*	Eudicots	Brassicales	30	41	9	20	^23^
9	*Arabis alpina*	Eudicots	Brassicales	9	9	0	0	–
10	*Boechera stricta*	Eudicots	Brassicales	30	35	5	10	–
11	*Brassica napus*	Eudicots	Brassicales	125	125	0	0	^30^
12	*Brassica oleracea*	Eudicots	Brassicales	82	82	0	0	–
13	*Brassica rapa*	Eudicots	Brassicales	79	79	0	0	–
14	*Camelina sativa*	Eudicots	Brassicales	94	94	0	0	–
15	*Capsella grandiflora*	Eudicots	Brassicales	28	29	1	2	–
16	*Capsella rubella*	Eudicots	Brassicales	35	35	0	0	–
17	*Eutrema salsugineum*	Eudicots	Brassicales	34	34	0	0	–
18	*Raphanus raphanistrum*	Eudicots	Brassicales	57	57	0	0	–
19	*Raphanus sativus*	Eudicots	Brassicales	59	59	0	0	–
20	*Sisymbrium irio*	Eudicots	Brassicales	28	28	0	0	–
21	*Thellungiella parvula*	Eudicots	Brassicales	31	31	0	0	–
22	*Carica papaya*	Eudicots	Brassicales	23	23	0	0	–
23	*Tarenaya hassleriana*	Eudicots	Brassicales	58	58	0	0	–
24	*Amaranthus hypochondriacus*	Eudicots	Caryophyllales	25	25	0	0	–
25	*Beta vulgaris*	Eudicots	Caryophyllales	16	18	2	4	–
26	*Dianthus caryophyllus*	Eudicots	Caryophyllales	25	25	0	0	–
27	*Spinacia oleracea*	Eudicots	Caryophyllales	16	20	3	7	–
28	*Citrullus lanatus*	Eudicots	Cucurbitales	24	24	0	0	–
29	*Cucumis melo*	Eudicots	Cucurbitales	25	30	4	9	^31^
30	*Cucumis sativus*	Eudicots	Cucurbitales	25	36	4	15	–
31	*Actinidia chinensis*	Eudicots	Ericales	40	40	0	0	–
32	*Arachis hypogaea*	Eudicots	Fabales	20	20	0	0	–
33	*Arachis duranensis*	Eudicots	Fabales	22	22	0	0	–
34	*Arachis ipaensis*	Eudicots	Fabales	22	22	0	0	–
35	*Cicer arietinum*	Eudicots	Fabales	33	33	0	0	^32^
36	*Glycine max*	Eudicots	Fabales	63	92	14	43	^26^
37	*Glycine soja*	Eudicots	Fabales	44	44	0	0	–
38	*Medicago truncatula*	Eudicots	Fabales	43	53	6	16	–
39	*Phaseolus vulgaris*	Eudicots	Fabales	32	35	3	6	^33^
40	*Trifolium pratense*	Eudicots	Fabales	33	33	0	0	–
41	*Vigna angularis*	Eudicots	Fabales	28	40	7	19	–
42	*Vigna radiata*	Eudicots	Fabales	24	24	0	0	–
43	*Vigna unguiculata*	Eudicots	Fabales	11	11	0	0	–
44	*Cajanus cajan*	Eudicots	Fabales	33	33	0	0	**–**
45	*Lotus japonicus*	Eudicots	Fabales	23	27	3	7	**–**
46	*Castanea mollissima*	Eudicots	Fagales	19	22	1	4	**–**
47	*Juglans regia*	Eudicots	Fagales	20	20	0	0	**–**
48	*Catharanthus roseus*	Eudicots	Gentianales	21	49	10	38	**–**
49	*Coffea canephora*	Eudicots	Gentianales	22	22	0	0	**–**
50	*Dorcoceras hygrometricum*	Eudicots	Lamiales	20	20	0	0	**–**
51	*Ocimum tenuiflorum*	Eudicots	Lamiales	25	25	0	0	**–**
52	*Salvia miltiorrhiza*	Eudicots	Lamiales	23	23	0	0	^34^
53	*Genlisea aurea*	Eudicots	Lamiales	18	18	0	0	**–**
54	*Utricularia gibba*	Eudicots	Lamiales	29	29	0	0	**–**
55	*Sesamum indicum*	Eudicots	Lamiales	36	36	0	0	**–**
56	*Mimulus guttatus*	Eudicots	Lamiales	26	36	8	18	**–**
57	*Jatropha curcas*	Eudicots	Malpighiales	28	28	0	0	**–**
58	*Manihot esculenta*	Eudicots	Malpighiales	36	46	7	17	^35^
59	*Ricinus communis*	Eudicots	Malpighiales	19	19	0	0	^36^
60	*Linum usitatissimum*	Eudicots	Malpighiales	35	35	0	0	**–**
61	*Populus euphratica*	Eudicots	Malpighiales	39	39	0	0	^21^
62	*Populus trichocarpa*	Eudicots	Malpighiales	39	76	19	56	^37^
63	*Salix purpurea*	Eudicots	Malpighiales	39	63	16	40	**–**
64	*Gossypium arboreum*	Eudicots	Malvales	46	46	0	0	^38^
65	*Gossypium hirsutum*	Eudicots	Malvales	88	88	0	0	^38^
66	*Gossypium raimondii*	Eudicots	Malvales	46	87	20	61	^38^
67	*Theobroma cacao*	Eudicots	Malvales	24	36	4	16	^39^
68	*Eucalyptus camaldulensis*	Eudicots	Myrtales	21	21	0	0	**–**
69	*Eucalyptus grandis*	Eudicots	Myrtales	23	30	5	12	**–**
70	*Nelumbo nucifera*	Eudicots	Proteales	34	34	0	0	**–**
71	*Aquilegia coerulea*	Eudicots	Ranunculales	28	47	6	25	**–**
72	*Cannabis sativa*	Eudicots	Rosales	16	21	2	7	**–**
73	*Humulus lupulus*	Eudicots	Rosales	18	18	0	0	**–**
74	*Morus notabilis*	Eudicots	Rosales	23	23	0	0	**–**
75	*Ziziphus jujuba*	Eudicots	Rosales	30	30	0	0	**–**
76	*Fragaria vesca*	Eudicots	Rosales	19	19	0	0	**–**
77	*Fragaria x ananassa*	Eudicots	Rosales	14	14	0	0	**–**
78	*Malus domestica*	Eudicots	Rosales	35	35	0	0	^40^
79	*Prunus mume*	Eudicots	Rosales	28	28	0	0	**–**
80	*Prunus persica*	Eudicots	Rosales	20	31	6	17	**–**
81	*Pyrus bretschneideri*	Eudicots	Rosales	32	32	0	0	**–**
82	*Azadirachta indica*	Eudicots	Sapindales	28	28	0	0	**–**
83	*Citrus clementina*	Eudicots	Sapindales	40	40	0	0	**–**
84	*Citrus sinensis*	Eudicots	Sapindales	55	55	0	0	^41^
85	*Kalanchoe laxiflora*	Eudicots	Saxifragales	37	60	16	39	**–**
86	*Kalanchoe marnieriana*	Eudicots	Saxifragales	72	100	18	46	**–**
87	*Ipomoea trifida*	Eudicots	Solanales	38	38	0	0	**–**
88	*Capsicum annuum*	Eudicots	Solanales	28	28	0	0	^42^
89	*Nicotiana benthamiana*	Eudicots	Solanales	59	59	0	0	**–**
90	*Nicotiana sylvestris*	Eudicots	Solanales	38	38	0	0	**–**
91	*Nicotiana tabacum*	Eudicots	Solanales	82	82	0	0	**–**
92	*Nicotiana tomentosiformis*	Eudicots	Solanales	44	44	0	0	**–**
93	*Petunia axillaris*	Eudicots	Solanales	30	30	0	0	**–**
94	*Petunia inflata*	Eudicots	Solanales	34	34	0	0	**–**
95	*Solanum lycopersicum*	Eudicots	Solanales	30	30	0	0	^43^
96	*Solanum melongena*	Eudicots	Solanales	26	26	0	0	^44^
97	*Solanum pennellii*	Eudicots	Solanales	33	37	4	8	**–**
98	*Solanum pimpinellifolium*	Eudicots	Solanales	30	30	0	0	**–**
99	*Solanum tuberosum*	Eudicots	Solanales	50	50	0	0	^45^
100	*Vitis vinifera*	Eudicots	Vitales	19	19	0	0	^46,47^
101	*Spirodela polyrhiza*	Monocots	Alismatales	20	20	0	0	**–**
102	*Zostera marina*	Monocots	Alismatales	24	24	0	0	**–**
103	*Elaeis guineensis*	Monocots	Arecales	42	42	0	0	**–**
104	*Phoenix dactylifera*	Monocots	Arecales	19	19	0	0	**–**
105	*Phalaenopsis equestris*	Monocots	Asparagales	20	20	0	0	**–**
106	*Ananas comosus*	Monocots	Poales	18	18	0	0	**–**
107	*Aegilops tauschii*	Monocots	Poales	18	18	0	0	**–**
108	*Brachypodium distachyon*	Monocots	Poales	29	34	5	10	^48^
109	*Brachypodium stacei*	Monocots	Poales	28	32	2	6	**–**
110	*Dichanthelium oligosanthes*	Monocots	Poales	33	33	0	0	**–**
111	*Eragrostis tef*	Monocots	Poales	23	23	0	0	**–**
112	*Hordeum vulgare*	Monocots	Poales	16	32	9	25	**–**
113	*Leersia perrieri*	Monocots	Poales	25	33	4	12	**–**
114	*Oropetium thomaeum*	Monocots	Poales	13	13	0	0	**–**
115	*Oryza barthii*	Monocots	Poales	23	23	0	0	**–**
116	*Oryza brachyantha*	Monocots	Poales	21	21	0	0	**–**
117	*Oryza glaberrima*	Monocots	Poales	23	23	0	0	**–**
118	*Oryza glumaepatula*	Monocots	Poales	27	32	4	9	**–**
119	*Oryza longistaminata*	Monocots	Poales	13	13	0	0	**–**
120	*Oryza meridionalis*	Monocots	Poales	23	24	1	2	**–**
121	*Oryza nivara*	Monocots	Poales	26	31	4	9	**–**
122	*Oryza punctata*	Monocots	Poales	30	34	3	7	**–**
123	*Oryza rufipogon*	Monocots	Poales	28	32	4	8	**–**
124	*Oryza sativa* subsp.* indica*	Monocots	Poales	31	31	0	0	^23^
125	*Oryza sativa* subsp.* japonica*	Monocots	Poales	26	32	6	12	^23^
126	*Panicum hallii*	Monocots	Poales	31	44	8	21	**–**
127	*Panicum virgatum*	Monocots	Poales	66	104	21	59	**–**
128	*Phyllostachys heterocycla*	Monocots	Poales	31	31	0	0	**–**
129	*Saccharum officinarum*	Monocots	Poales	6	6	0	0	–
130	*Setaria italica*	Monocots	Poales	31	37	6	12	^49^
131	*Setaria viridis*	Monocots	Poales	31	39	6	14	**–**
132	*Sorghum bicolor*	Monocots	Poales	31	40	7	16	^50^
133	*Triticum aestivum*	Monocots	Poales	48	48	0	0	^51–53^
134	*Triticum urartu*	Monocots	Poales	17	17	0	0	**–**
135	*Zea mays*	Monocots	Poales	38	54	10	26	^54^
136	*Zoysia japonica*	Monocots	Poales	39	39	0	0	**–**
137	*Zoysia matrella*	Monocots	Poales	73	73	0	0	**–**
138	*Zoysia pacifica*	Monocots	Poales	50	50	0	0	**–**
139	*Musa acuminata*	Monocots	Zingiberales	51	51	0	0	**–**
140	*Amborella trichopoda*	Basal angiosperm	Amborellales	20	20	0	0	**–**
141	*Picea abies*	Gymnosperm	-	9	9	0	0	**–**
142	*Picea glauca*	Gymnosperm	-	6	6	0	0	**–**
143	*Picea sitchensis*	Gymnosperm	-	4	4	0	0	**–**
144	*Pinus taeda*	Gymnosperm	-	5	5	0	0	**–**
145	*Pseudotsuga menziesii*	Gymnosperm	-	19	19	0	0	**–**
146	*Marchantia polymorpha*	Marchantiophyta	-	6	10	1	5	**–**
147	*Physcomitrella patens*	Bryophyta	-	15	53	15	53	–
148	*Sphagnum fallax*	Bryophyta	-	15	35	6	26	**–**
149	*Selaginella moellendorffii*	Lycopodiophyta	-	8	8	0	0	**–**
150	*Klebsormidium flaccidum*	Charophyta	-	10	10	0	0	**–**
151	*Auxenochlorella protothecoides*	Chlorophytae	-	6	6	0	0	**–**
152	*Bathycoccus prasinos*	Chlorophytae	-	7	7	0	0	**–**
153	*Chlamydomonas reinhardtii*	Chlorophytae	-	15	15	0	0	^55^
154	*Chlorella variabilis NC64A*	Chlorophytae	-	8	8	0	0	**–**
155	*Coccomyxa subellipsoidea C-169*	Chlorophytae	-	6	6	0	0	**–**
156	*Dunaliella salina*	Chlorophytae	-	8	9	1	2	**–**
157	*Gonium pectorale*	Chlorophytae	-	7	7	0	0	**–**
158	*Micromonas pusilla CCMP1545*	Chlorophytae	-	10	10	0	0	**–**
159	*Micromonas sp. RCC299*	Chlorophytae	-	8	8	0	0	**–**
160	*Monoraphidium neglectum*	Chlorophytae	-	9	9	0	0	**–**
161	*Ostreococcus lucimarinus*	Chlorophytae	-	8	8	0	0	**–**
162	*Ostreococcus sp. RCC809*	Chlorophytae	-	5	5	0	0	**–**
163	*Ostreococcus tauri*	Chlorophytae	-	5	5	0	0	**–**
164	*Picochlorum sp. SENEW3*	Chlorophytae	-	6	6	0	0	**–**
165	*Volvox carteri*	Chlorophytae	-	13	15	2	4	**–**
**Total**				**4,762**	**5,335**	**329**	**902**	**–**

**Fig. 1:**
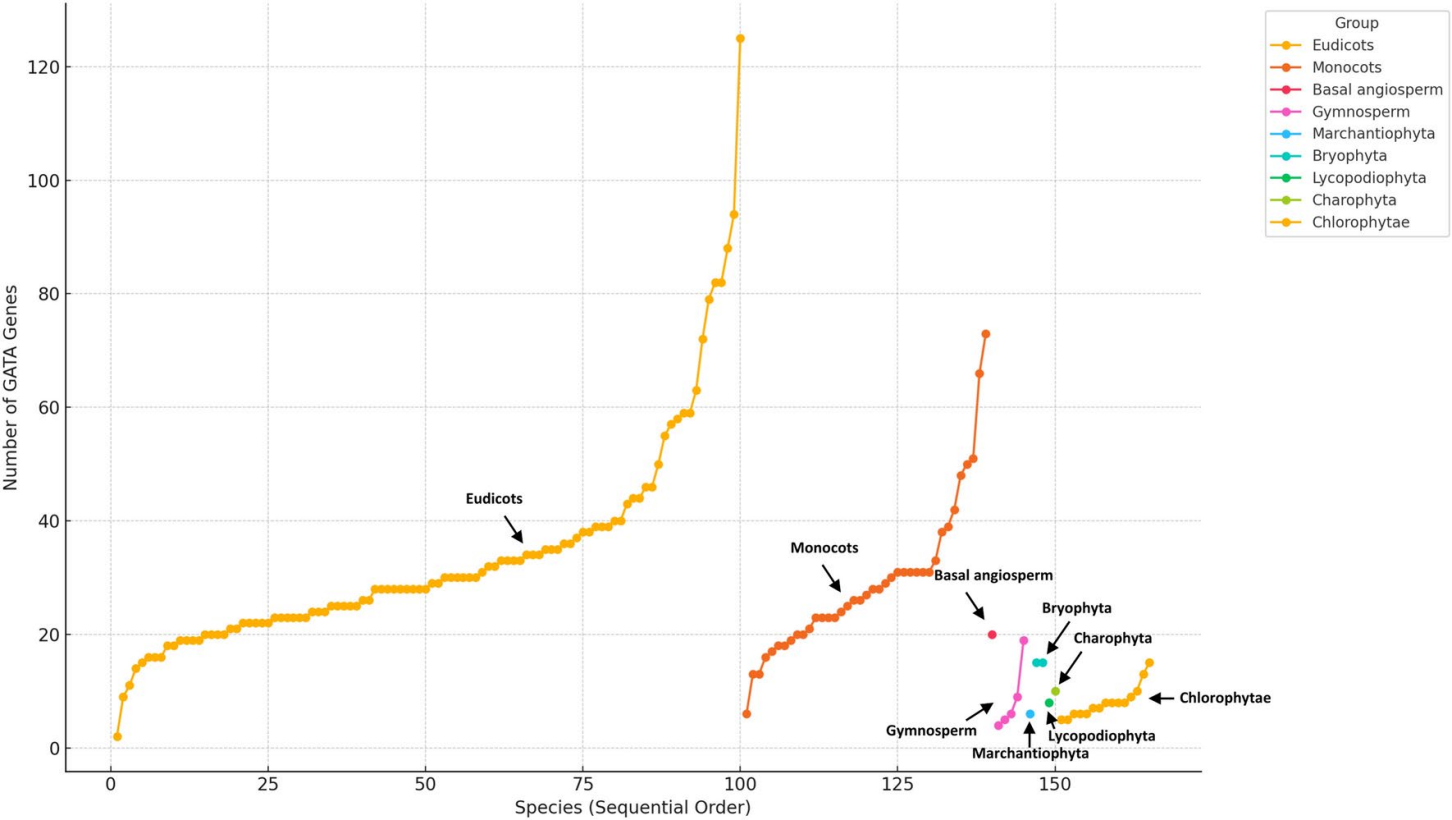
Distribution of GATA gene numbers across different plant groups. The plot illustrates the number of GATA genes in various plant species arranged in sequential order, grouped by major plant classifications. The x-axis represents species in a sequential order, while the y-axis shows the number of GATA genes identified in each species. Each point represents a species, and lines connect species within the same group. Different colors denote plant groups: eudicots (yellow), monocots (orange), basal angiosperms (red), gymnosperms (magenta), marchantiophyta (cyan), bryophyta (green), lycopodiophyta (blue), charophyta (dark green), and chlorophyta (gold). Notable groups are labeled, including “eudicots” and “monocots”, highlighting their gene distribution patterns within the phylogenetic context.

The analysis of plant GATA genes revealed substantial variability in gene numbers among different taxonomic groups (Fig. 1). In angiosperm lineages, particularly eudicots and monocots, the number of GATA genes was relatively high compared to non-angiosperm groups. Eudicots displayed a wide range, from 2 genes in *Helianthus annuus* to 125 in *Brassica napus*, with an average of approximately 33.82 genes per species. Monocots exhibited a range from 6 genes in *Saccharum officinarum* to 73 in *Zoysia matrella*, averaging around 29.28 genes per species. A significant proportion of angiosperm species (112 out of 139; 80.58%) had between 15 and 40 GATA genes, suggesting a common range for GATA gene numbers across angiosperms. In contrast, non-angiosperm groups, such as bryophyta, charophyta, and chlorophytae, possessed fewer GATA genes, typically ranging from 4 to 20, indicating a more conserved gene number in these lineages. This expanded analysis, including additional plant groups, provides insight into the evolutionary expansion and functional diversification of GATA TFs across the plant kingdom, establishing a valuable foundation for further functional and evolutionary studies.

### Variation in amino acid sequence lengths of plant GATA TFs

**Fig. 2:**
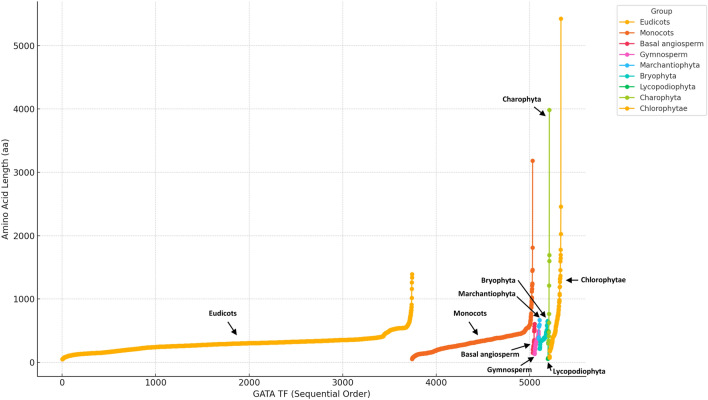
Distribution of amino acid lengths of GATA TFs across plant groups. The plot shows the amino acid length of GATA TFs for various plant species arranged in sequential order. The x-axis represents individual GATA TFs in a sequential order, while the y-axis displays their amino acid length (aa). Each point corresponds to a GATA TF from a specific species, and points are color-coded according to plant groups: eudicots (yellow), monocots (orange), basal angiosperms (red), gymnosperms (magenta), marchantiophyta (cyan), bryophyta (green), lycopodiophyta (blue), charophyta (dark green), and chlorophyta (gold). Labels identify major plant groups, including “eudicots” and “charophyta”, highlighting the variability in GATA TF lengths within and across lineages.

Based on the amino acid sequence lengths of GATA TFs across various plant groups, I observed considerable diversity in sequence length, suggesting evolutionary adaptation to different functional roles (Fig. 2). In eudicots, GATA TFs ranged from 47 amino acids (*icon00013032* in *Fragaria* x *ananassa*) to 1,392 amino acids (*rscf00000768* in *Fragaria* x *ananassa*), with an average length of 293.60 amino acids. Monocots exhibited a range from 50 amino acids (*EMT24406* in *Aegilops tauschii*) to 3,185 amino acids (*KN540155.1* in *Oryza longistaminata*), averaging 318.12 amino acids. These groups showed a concentration of sequence lengths between 100 and 400 amino acids, with 85.53% (4,304 out of 5,032) of GATA TFs in this range, highlighting a common functional length in angiosperms.

For *Amborella trichopoda*, a basal angiosperm, the sequence lengths ranged from 151 to 608 amino acids, with an average of 321.35 amino acids, suggesting a possible intermediary length distribution between eudicots and monocots. In contrast, non-angiosperm groups such as bryophyta, charophyta, and chlorophytae displayed a trend toward longer sequences, with 133 out of 283 GATA TFs (approximately 47%) exceeding 400 amino acids, resulting in a higher average length of 524.97 amino acids. This pattern indicates that non-angiosperms may retain longer GATA TF sequences, potentially reflecting structural or functional features that have been conserved for specific roles in these lineages, rather than undergoing the length diversification seen in angiosperms.

The observed diversity in amino acid sequence lengths within and across plant groups reflects evolutionary pressures that have shaped GATA TFs to meet distinct functional needs. These findings suggest that, while angiosperms have evolved more compact and diverse GATA TF structures, non-angiosperms retain longer sequences, which may correspond to conserved functions. This analysis provides a foundation for further studies on the functional implications of GATA TF length variation across phylogenetic groups.

### Genus-level variation of plant GATA genes

The analysis of GATA gene distribution across various plant species within the same genus highlights significant evolutionary trends in gene diversification and specialization among plants. Notably, 76 out of 165 plant species belong to 27 genera, including well-studied genera such as *Arabidopsis*, *Brassica*, *Gossypium*, *Oryza*, *Solanum*, and *Nicotiana* (Table S2). Each genus exhibits a unique range of GATA gene numbers, suggesting evolutionary pressures that may have influenced gene gain or loss events.

For example, within the genus *Arabidopsis*, GATA gene numbers vary from 22 in *Arabidopsis halleri* to 30 in *A. thaliana*. Such variation may reflect adaptive responses to different environmental conditions or developmental needs, allowing *Arabidopsis* species to optimize functions like chloroplast development and environmental response mechanisms. In *Oryza*, the number of GATA genes ranges from 13 in *Oryza longistaminata* to 31 in *Oryza sativa* subsp. *indica*, again indicating potential adaptation to diverse ecological niches. Moreover, species in the *Brassica* genus display the highest observed GATA gene count, with *Brassica napus* possessing 125 GATA genes. This exceptionally high number of GATA genes in *B. napus* suggests extensive gene duplication events, possibly driven by the need to support complex regulatory networks for environmental resilience and growth optimization. Similar trends of gene number variation are also observed in genera such as *Gossypium* and *Nicotiana*, further reinforcing the notion that GATA gene diversification plays a critical role in plant adaptability and functional specialization.

These findings underscore the evolutionary importance of GATA gene variation within genera, reflecting both gene duplication and functional specialization in response to environmental and developmental demands. This genus-level analysis of GATA TF distribution provides a foundation for understanding how these TFs contribute to the ecological adaptability of plants.

### Impact of alternative splicing on functional diversity of plant GATA genes

Alternative splicing (AS), a process that enables a single gene to produce multiple protein isoforms by rearranging exon and intron sequences, was observed in 49 out of 165 plant species (29.70%), including 28 species in eudicots and 16 species in monocots exhibiting AS forms of the GATA gene (Table 2 and Table S3). Across these species, a total of 329 GATA genes (204 in eudicots, 100 in monocots, 1 in marchantiophyta, 21 in bryophyta, and 3 in chlorophytae) had AS forms, generating 902 distinct AS variants. The majority of these GATA genes (302 out of 329; 91.79%) exhibited 2 to 4 AS forms, though some displayed up to 12 distinct AS forms, such as *Aqcoe5G315100* from *Aquilegia coerulea*, highlighting the potential for extensive functional diversification.

**Table 2:** Number of AS forms of plant GATA genes.

Number of AS forms	Number of plant GATA genes									Ratio of total GATA genes having AS form
	**Eudicots**	**Monocots**	**Basal angiosperm**	**Gymnosperm**	**Marchantio-phyta**	**Bryo-phyta**	**Lycopodio-phyta**	**Charo-phyta**	**Chloro-phytae**	
2	126	74	0	0	0	13	0	0	3	65.65%
3	44	14	0	0	0	0	0	0	0	17.63%
4	21	8	0	0	0	4	0	0	0	10.03%
5	2	2	0	0	1	1	0	0	0	1.82%
6	4	1	0	0	0	0	0	0	0	1.52%
7	2	0	0	0	0	0	0	0	0	0.61%
8	2	0	0	0	0	0	0	0	0	0.61%
9	0	0	0	0	0	0	0	0	0	0.00%
10	2	1	0	0	0	2	0	0	0	1.52%
11	0	0	0	0	0	0	0	0	0	0.00%
12	1	0	0	0	0	1	0	0	0	0.61%
**Total**	**204**	**100**	**0**	**0**	**1**	**21**	**0**	**0**	**3**	**100.00%**

Among these 329 GATA genes, 75 in eudicots, 10 in monocots, 1 in marchantiophyta, 1 in chlorophytae, and 18 in bryophyta had AS forms that retained the same coding regions across variants, with sequence differences restricted to the 5’ and/or 3’ untranslated regions (UTRs). Notably, the majority of bryophyta GATA genes (18 out of 21) displayed this form of AS, where coding sequences remained identical, suggesting that AS in bryophyta may primarily modulate gene expression levels through UTR variations rather than altering the protein structure.

Further structural analyses of AS forms in model species like *Populus trichocarpa*^21^, *Arabidopsis thaliana*^22^, and *Oryza sativa*^20^ revealed that AS forms exhibited a range of exon differences in both coding regions and UTRs. These structural differences imply functional diversification among AS forms, as variations in exon composition can influence protein functionality, while UTR alterations may affect mRNA stability and translational efficiency. For instance, the *OsGATA23* gene in *O. sativa* has two AS forms, *OsGATA23a* and *OsGATA23b*, which show differential expression in response to salinity and drought stress, indicating that each AS variant may play distinct biological roles under stress conditions^20^. This highlights the importance of exploring AS forms in plant GATA genes to fully understand their functional implications, as AS serves as a versatile mechanism for expanding the regulatory capacity and adaptability of GATA TFs in response to environmental cues and developmental needs.

### Diversity and adaptation of plant GATA domains

The GATA domain, characterized by the presence of four cysteines that bind a zinc ion to form a zinc finger, is critical for binding to specific DNA sequences (WGATAR; W = T or A, R = G or A). Most GATA TFs contain a single GATA domain, although some, such as *OsGATA24, 25, and 26* in *O. sativa*^23^, possess two or more GATA domains. Based on the number of amino acids between the second and third cysteines of the GATA domain, three primary types have been identified: type IVa (CX_2_CX_17_CX_2_C), typically found in mammals^24^ and fungi^25^; type IVb (CX_2_CX_18_CX_2_C), common in plants^21,22^ and fungi^25^; and type IVc (CX_2_CX_20_CX_2_C), also found in plants^21,22^ and fungi^25^. Additionally, two unusual types have been noted: type IV4 (CX_4_CX_18_CX_2_C), which binds zinc molecules (e.g., *GmGATA50* in *Glycine max*^26^), and type IVp (partial type), such as *AtGATA26a* (CX_15_CX_2_C) in *A. thaliana*^22^ and *GmGATA28* (CX_2_CX_12_) in *Glycine max*^26^. The diversity in GATA domain types likely reflects adaptations to different environmental and developmental contexts, suggesting specialized regulatory mechanisms across species. A study on GATA family evolution shows that, while the DNA-binding domain is highly conserved, specific amino acid variations contribute to evolutionary and functional diversification^24^. This underscores the role of conserved residues in structural integrity and highlights the need for experimental validation to confirm functional roles associated with domain diversity.

5,536 GATA domains were identified across 5,335 plant GATA TFs, with each TF containing between one and five GATA domains. Most TFs contained a single GATA domain (5,184 out of 5,335; 97.17%), with fewer containing multiple domains: two domains (111 out of 5,335; 2.08%), three (32 out of 5,335; 0.60%), four (6 out of 5,335; 0.11%), and five (2 out of 5,335; 0.04%) (Table 3; Table S4). Monocots demonstrated a higher proportion of TFs with multiple domains (97 out of 1,290; 7.52%) compared to eudicots (46 out of 3,742; 1.23%). This suggests that monocots may have evolved more complex regulatory needs, possibly due to their unique ecological niches and adaptive strategies, requiring diversified transcriptional regulation.

**Table 3:** GATA domain types of plant GATA TFs.

Domain type	form	Number of plant GATA domains									Ratio of total GATA domain
		**Eudicots**	**Monocots**	**Basal angiosperm**	**Gymnosperm**	**Marchantio-phyta**	**Bryo-phyta**	**Lycopodio-phyta**	**Charo-phyta**	**Chloro-phytae**	
Type IVb	CX_2_CX_18_CX_2_C	2,867	1,079	15	33	7	36	3	8	83	74.62%
Type IVc	CX_2_CX_20_CX_2_C	642	241	2	6	2	48	1	1	7	17.16%
Type IVp	Partial	224	81	3	4	0	0	2	1	8	5.83%
Type IV4	CX_4_CX_18_CX_2_C	40	0	0	0	0	0	0	0	0	0.72%
Type IVa	CX_2_CX_17_CX_2_C	26	0	0	0	0	0	0	0	0	0.47%
Type IVe	CX_2_CX_-16,21-_CX_2_C	12	11	0	0	1	4	2	3	33	1.19%
**Total**		**3,811**	**1,412**	**20**	**43**	**10**	**88**	**8**	**13**	**131**	**100.00%**

Among the GATA domain types, type IVb (CX_2_CX_18_CX_2_C) was the most prevalent, encompassing 74.62% (4,131 out of 5,536) of all GATA domains, followed by type IVc (CX_2_CX_20_CX_2_C) at 17.16% (950 out of 5,536). These two types dominate both eudicot and monocot lineages, reflecting strong evolutionary conservation and suggesting essential regulatory roles shared across angiosperms. Notably, type IVb was particularly abundant in eudicots and monocots, but was also present in non-angiosperm groups, including bryophyta and chlorophyta, indicating that this domain type may have originated early in plant evolution and retained critical functions across diverse plant groups.

Minor GATA domain types, (i) type IVp (partial), (ii) type IV4 (CX_4_CX_18_CX_2_C), (iii) type IVa (CX_2_CX_17_CX_2_C), and (iv) type IVe (CX_2_CX_-16,21-_CX_2_C), accounted for a combined 8.22% of GATA domains. Type IVp was the most common among these, with 323 instances, primarily in eudicots and monocots. This domain’s partial structure suggests potential regulatory modifications or evolutionary remnants with specific roles in angiosperms. Type IV4 and IVa, although rare, were primarily identified in eudicots and suggest niche functional roles, possibly adapted from similar domains found in fungi. The unique type IVe GATA domains, which vary in amino acid length between the second and third cysteine residues, were observed across multiple plant groups, including non-angiosperms like chlorophyta, highlighting functional diversification across lineages.

Non-angiosperms, such as bryophyta and chlorophyta, exhibited unique distribution patterns. For example, type IVb is still dominant, albeit at a lower prevalence than in angiosperms, suggesting that it retains fundamental roles in these lineages. Type IVc domains, while less common, were notably present in bryophyta, reinforcing the hypothesis that these domains have functional significance beyond angiosperm-specific regulatory roles. These findings highlight the evolutionary flexibility and adaptability of GATA domains across both ancient and modern plant lineages.

Additionally, AS events contributed to domain diversity, particularly in altering the structure from complete domains (types IVb and IVc) to partial forms (type IVp). For instance, AS events were observed in 28 plant GATA genes, either partially losing the domain or converting from type IVb to IVp or from type IVc to IVp (Table 4). This variability may influence GATA TF function by altering DNA-binding capacity or protein stability, thus providing a mechanism for regulatory diversification.

**Table 4:** GATA domain type change by AS forms in plant GATA TFs.

No	Species	Groups	GATA gene	Number of AS forms	Type IVb	Type IVc	Type IVp
1	*Arabidopsis thaliana*	Eudicots	*AT4G17570*	3	2	0	1
2	*Catharanthus roseus*	Eudicots	*cra_locus_11133*	4	3	0	1
3			*cra_locus_8285*	4	0	3	1
4	*Cucumis melo*	Eudicots	*MELO3C013904*	2	1	0	1
5	*Glycine max*	Eudicots	*07G243000*	2	1	0	1
6			*08G221800*	10	0	8	2
7	*Gossypium raimondii*	Eudicots	*009G031400*	2	1	0	1
8	*Hordeum vulgare*	Monocots	*MLOC_62522*	2	1	0	1
9	*Kalanchoe laxiflora*	Eudicots	*Kaladp0095s0409*	2	1	0	1
10			*Kaladp0500s0002*	2	1	0	1
11	*Kalanchoe marnieriana*	Eudicots	*0461s0023*	2	1	0	1
12	*Oryza rufipogon*	Monocots	*ORUFI01G16060*	2	1	0	1
13	*Oryza sativa* subsp. *japonica*	Monocots	*LOC_Os01g24070*	2	1	0	1
14	*Panicum virgatum*	Monocots	*5KG230300*	2	1	0	1
15			*5NG606200*	2	1	0	1
16			*7NG220500*	2	1	0	1
17	*Phaseolus vulgaris*	Eudicots	*009G118600*	2	0	1	1
18	*Populus trichocarpa*	Eudicots	*005G020500*	2	1	0	1
19			*006G229200*	2	1	0	1
20			*014G124400*	2	1	0	1
21			*017G042200*	5	0	4	1
22	*Salix purpurea*	Eudicots	*0086s0290*	2	1	0	1
23			*0201s0160*	3	2	0	1
24	*Setaria viridis*	Monocots	*7G157500*	3	1	0	2
25	*Sorghum bicolor*	Monocots	*009G050600*	2	1	0	1
26	*Vigna angularis*	Eudicots	*Vang0268s00180*	3	1	0	2
27			*Vang07g01810*	3	2	0	1
**Total**			**27**	**74**	**28**	**16**	**30**

In conclusion, the conserved distribution of types IVb and IVc across angiosperms and non-angiosperms underscores the critical regulatory roles these domains play. The presence of unique types (IV4, IVa, and IVe) across certain lineages, alongside AS-driven modifications, suggests an adaptive landscape where GATA TFs evolve to meet the distinct regulatory demands of each plant group. These findings enhance our understanding of the functional diversity and evolutionary adaptability of GATA TFs across the plant kingdom.

### Conservation and functional divergence of plant GATA domain types

**Fig. 3:**
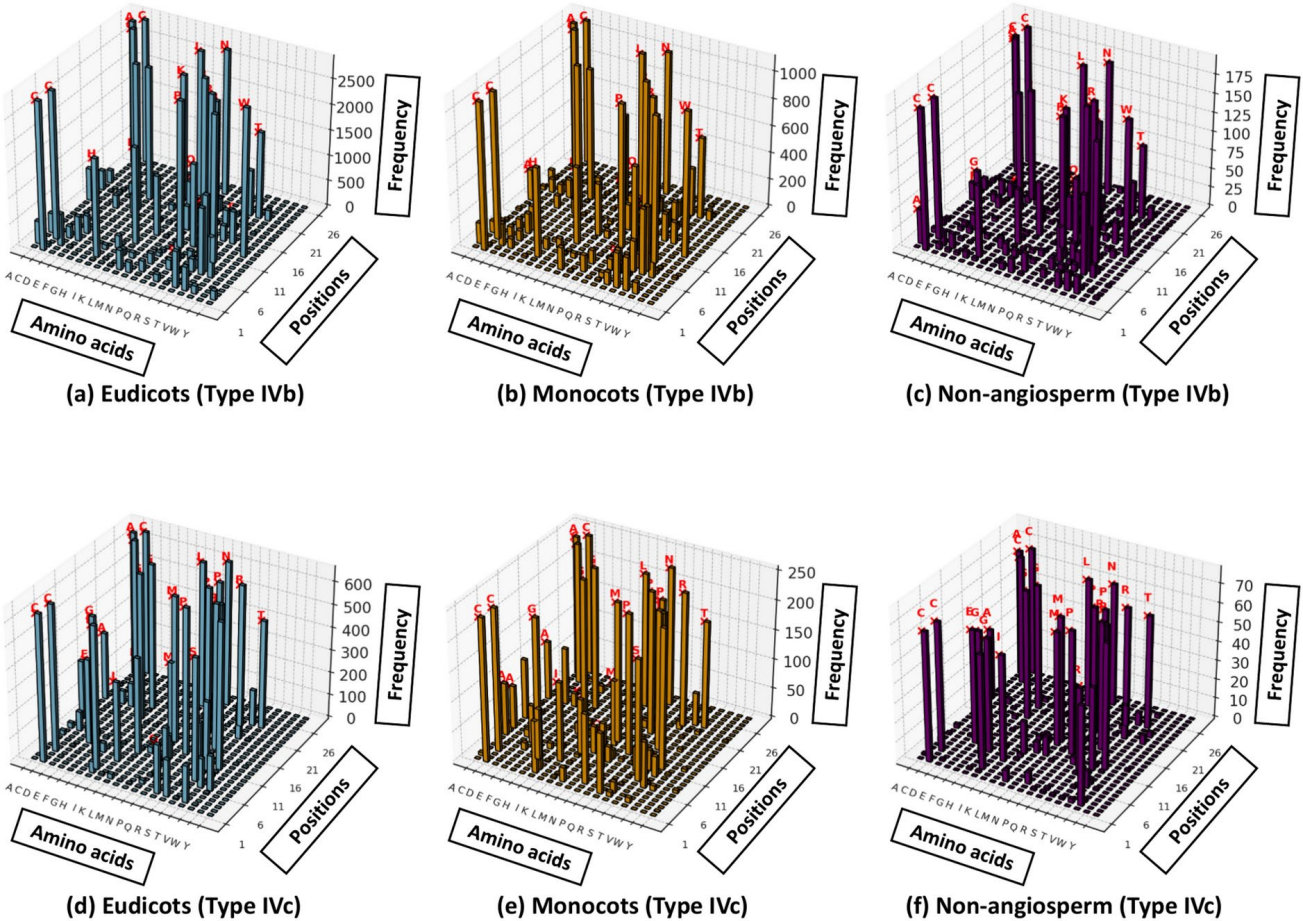
Amino acid frequency distribution across GATA domains in different plant groups. This figure shows 3D frequency plots of amino acid distribution at specific positions within type IVb and type IVc of GATA domains across different plant groups. Each subfigure represents a specific plant group and GATA domain type. The x-axis shows the amino acid types (A, C, D, E, etc.), the y-axis represents amino acid positions (1–26 or 1–28) within the GATA TF sequence, and the z-axis indicates the frequency of each amino acid at each position. (**a**) shows the amino acid sequence patterns of type IVb in eudicots, (**b**) shows type IVb in monocots, (**c**) shows type IVb in non-angiosperms, (**d**) shows type IVc in eudicots, (**e**) shows type IVc in monocots, and (**f**) shows type IVc in non-angiosperms. Red labels mark amino acids with notably high frequencies.

In type IVb, the most prevalent GATA domain type, four cysteine residues (C-1, C-4, C-23, and C-26) are completely conserved across eudicots, monocots, and non-angiosperms (Fig. 3a,b,c). These residues are critical for zinc finger formation, a structural feature essential for DNA binding. Additionally, residues such as T-9, P-10, W-12, R-13, G-15, P-16, G-18, L-22, N-24, and A-25 are highly conserved (> 90%) in both eudicots and monocots, underscoring their functional importance in stabilizing DNA–protein interactions and maintaining protein integrity. Interestingly, non-angiosperms show slightly reduced conservation levels for some of these residues, such as lower frequencies of T-7 and K-8, which may suggest adaptive divergence in DNA-binding specificity and protein stability. These subtle differences across plant groups imply that while the core function of type IVb GATA domains is preserved, there may be nuanced functional adaptations suited to each group’s unique regulatory requirements.

Type IVc, the second most common GATA domain type, exhibits full conservation of six key residues (C-1, C-4, P-18, G-20, C-25, and C-28) across eudicots, monocots, and non-angiosperms, reinforcing the structural stability of the zinc finger motif in this domain type (Fig. 3d,e,f). Additional residues, including G-5, T-11, P-12, M-14, R-15, R-16, G-17, P-18, G-20, R-22, L-24, N-26 and A-27, are highly conserved in eudicots and monocots (> 90%), suggesting that these positions play a significant role in enhancing DNA-binding affinity and specificity. Notably, at Position 2, the non-angiosperm group shows a high prevalence of the amino acid V (approximately 76%), while Monocots and Eudicots predominantly contain Q at this position, highlighting a distinctive divergence that may relate to functional adaptations in non-angiosperms. Similarly, at position 6, approximately 80% of non-angiosperms have the amino acid I, whereas monocots and eudicots display a lower conservation at this position, with only around 50% containing I. These unique patterns in non-angiosperms suggest evolutionary adaptations that may cater to regulatory requirements distinct from those in angiosperm lineages.

Types IV4, IVa, and IVe domains display distinct conserved amino acid patterns essential for maintaining the structural and functional integrity of the GATA domain. These patterns include specific residues critical for zinc ion coordination and DNA binding, underscoring their evolutionary significance in regulatory processes.

The observed conservation of specific residues within GATA domain across eudicots and monocots suggests a strong selection pressure to maintain GATA domain functionality in these groups, possibly due to shared regulatory roles in growth and development. In contrast, the divergence observed in non-angiosperms highlights potential adaptations to differing environmental or physiological conditions, which may have shaped the GATA domains to support unique regulatory needs. These findings underscore the evolutionary flexibility of GATA domains, with highly conserved core residues for essential functions and variable regions that enable functional diversity, particularly in non-angiosperm lineages.

### Taxonomic distribution and evolutionary trends of plant GATA genes

**Fig. 4:**
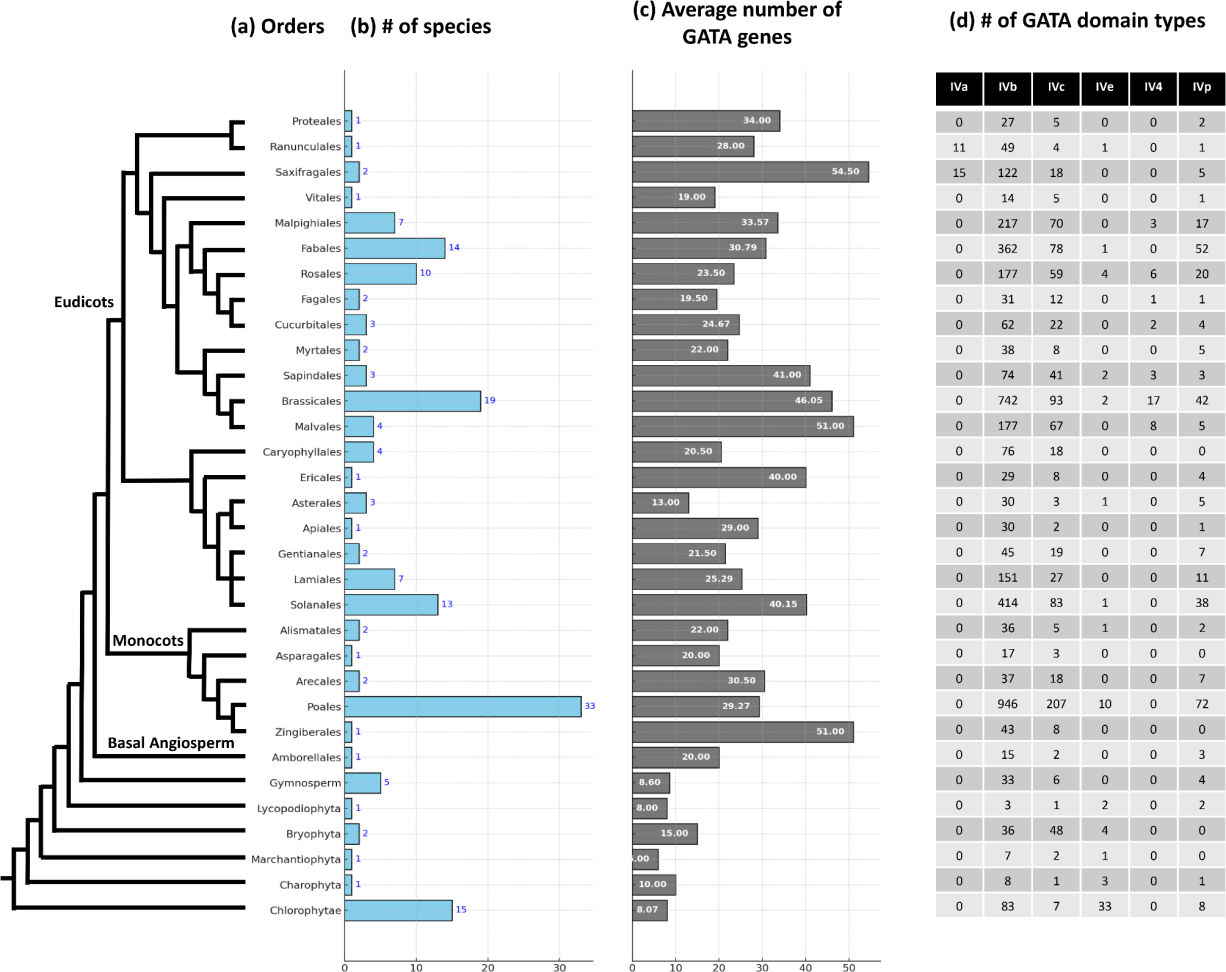
Phylogenetic distribution and characteristics of GATA genes and domain types across plant orders. The figure displays the distribution and characteristics of GATA genes and domain types across various plant orders. (**a**) The phylogenetic tree illustrates the evolutionary relationships among the major plant groups, including eudicots, monocots, basal angiosperms, and gymnosperms. (**b**) The bar graph shows the number of species sampled within each order. (**c**) The average number of GATA genes per species in each order is presented, with values annotated on the bars. (**d**) The table lists the distribution of GATA domain types (IVa, IVb, IVc, IVe, IV4, and IVp) for each order, providing insight into the diversity of GATA domains within and across groups.

The taxonomic distribution of GATA genes across plant lineages, including eudicots, monocots, and non-angiosperms, provides valuable insights into their evolutionary history and functional diversity. In this study, I analyzed a comprehensive dataset encompassing 20 eudicot orders, 5 monocot orders, and multiple non-angiosperm groups, such as gymnosperms, bryophyta, and chlorophyta (Fig. 4).

Eudicots display significant variation in the number of GATA genes across orders, ranging from 1 to 19 species per order, with average GATA gene counts spanning from 19.00 in vitales to 54.50 in saxifragales. Monocots exhibit a similar pattern, with species counts per order varying from 1 to 33, and average GATA gene numbers from 20.00 in asparagales to 51.00 in zingiberales. These variations in GATA gene abundance across angiosperm orders suggest adaptive diversification, potentially in response to the unique ecological and physiological demands of each lineage.

Among GATA domain types, type IVb is the most prevalent across both eudicots and monocots, indicating its evolutionary conservation and fundamental regulatory role in angiosperms. Following type IVb, type IVc ranks as the second most abundant domain type across nearly all angiosperm orders, reinforcing its functional importance in plant development and stress responses. Type IVp also appears frequently in most angiosperm orders, with the exception of caryophyllales, asparagales, and zingiberales, suggesting a role that may be conserved across various angiosperm lineages but less critical in these specific groups. Type IVa, on the other hand, is restricted to ranunculales and saxifragales, implying a specialized function or unique evolutionary pathway within these eudicot orders.

Distinct patterns emerge when examining the distribution of less common GATA domain types. Type IV4, found exclusively in eudicots, spans seven orders (malpighiales, rosales, fagales, cucurbitales, sapindales, brassicales, and malvales). This specific distribution pattern suggests a unique functional role within the eudicot lineage. Additionally, type IVe, which appears in seven orders across both eudicots and monocots (including ranunculales, rosales, sapindales, brassicales, solanales, alismatales, and poales), indicates a broader functional relevance extending across major angiosperm lineages.

The inclusion of non-angiosperm groups further reveals evolutionary nuances. Gymnosperms, bryophyta, lycopodiophyta, and chlorophyta exhibit lower overall GATA gene counts compared to angiosperms, with type IVb remaining the dominant domain type, though at a reduced frequency. This suggests that type IVb GATA domains have retained essential regulatory functions even in early-diverging plant lineages. Notably, type IVc also appears in bryophyta and chlorophyta, reinforcing the evolutionary significance of this domain type across both vascular and non-vascular plants.

In summary, the distribution of GATA domain types across angiosperms and non-angiosperms highlights both conservation and diversification within this GATA TF family. The high prevalence of types IVb and IVc across diverse lineages underscores their fundamental roles in plant regulatory networks. Meanwhile, the more restricted distribution of types IVa, IV4, and IVe points to lineage-specific adaptations that may reflect distinct regulatory requirements or environmental pressures. This comprehensive view enhances our understanding of the functional evolution of GATA TFs in the plant kingdom.

## Conclusion

This study provides a comprehensive analysis of GATA TFs across both angiosperm and non-angiosperm plants, shedding light on the evolutionary significance and functional diversity of this gene family. By examining conserved and variable amino acid residues, I reveal how GATA TFs maintain structural integrity through conserved residues while allowing functional flexibility through variable positions. The pervasive presence of type IVb and type IVc domains across diverse plant lineages highlights their fundamental roles in regulatory processes, emphasizing their evolutionary importance from ancient non-angiosperms to more derived angiosperms.

My findings underscore the lineage-specific adaptations within GATA TFs, with certain domain types (e.g., type IVa, IV4, and IVe) exhibiting more restricted distribution patterns, suggesting unique evolutionary paths and potential specialized functions in specific plant groups. This broad taxonomic distribution emphasizes the dynamic evolution of GATA domains, with structural conservation balanced by adaptations to meet distinct regulatory requirements across various plant groups.

With over 4,000 plant genomes now publicly available in databases like NCBI, future genome-wide studies on GATA TFs across this extensive dataset could yield even more detailed insights into the diversity and function of these TFs^27^. Comprehensive analysis of GATA TFs across a larger spectrum of plant genomes would refine my understanding of their roles in plant development, stress responses, and adaptation. Such research holds significant potential for agricultural and environmental sustainability, as it could inform breeding and genetic engineering strategies for developing stress-resistant crops that are better adapted to challenging environmental conditions, such as drought, salinity, and temperature extremes^28^. Leveraging insights from GATA TF diversity could pave the way for innovations in crop resilience and productivity, contributing to sustainable agricultural practices and food security.

This study lays the groundwork for further exploration into biological functions and ecological roles of GATA TFs, enriching my understanding of plant regulatory networks and their impact on plant survival and adaptation across evolutionary time. By linking GATA TFs to tangible applications in crop improvement, this research underscores the relevance of GATA TFs in applied plant science and biotechnology.

## Materials and methods

### Data collection and plant GATA genes

A comprehensive dataset of GATA genes in plant species was compiled using the PlantTFDB database (http://planttfdb.gao-lab.org/;^8^). This database was chosen due to its extensive coverage of TFs across diverse plant lineages. The analysis included a total of 140 species spanning both angiosperms (eudicots, monocots, and basal angiosperms) and 25 non-angiosperms, including representatives from gymnosperms, bryophyta, lycopodiophyta, and chlorophyta. The dataset encompassed species-specific identifiers, genomic sequences, and annotation details necessary for a comprehensive analysis of GATA TFs across both vascular and non-vascular plants. This inclusive approach provided a broad taxonomic perspective on the evolutionary diversity and functional conservation of GATA genes.

### Bioinformatic analysis

Bioinformatic tools facilitated the identification and annotation of GATA genes within the genomic sequences sourced from the PlantTFDB database. This involved rigorous sequence alignment, motif identification, particularly focusing on the GATA domain, and comprehensive verification against established TF databases. These processes ensured the accuracy and completeness of the dataset, laying the groundwork for subsequent analyses of GATA gene distribution, diversity, and evolutionary relationships across plant species.

### Alignment and analysis of GATA domain

The alignment of plant GATA domains utilized MEGA-X software^29^ (version 11) with the ClustalW alignment method. This approach systematically compared and aligned GATA domain sequences across the diverse plant species analyzed, enabling the identification of conserved regions and variable positions within the domains. This methodology provided insights into the sequence patterns and evolutionary relationships of GATA TFs among plant species.

### Characterization of GATA TFs

For each identified GATA gene, amino acid sequences of corresponding GATA TFs were extracted and analyzed. Sequence lengths, domain architectures (including the number and type of GATA domains), and structural variations due to alternative splicing were cataloged. Statistical analyses were conducted to determine average sequence lengths, domain compositions, and distribution patterns across different plant taxa.

### Taxonomic and phylogenetic analyses

Taxonomic information for each angiosperm species was curated to classify them into respective orders. Phylogenetic relationships among species were inferred based on established angiosperm phylogeny classification of flowering plants (APG-IV). This facilitated the interpretation of evolutionary trends and diversification patterns of GATA genes within the context of plant phylogeny.

### Statistical analysis

Statistical methods, including descriptive statistics and graphical representations, were employed to analyze the distribution patterns of GATA genes and their associated TFs across different taxa. This included calculating averages, ranges, and frequencies of GATA gene numbers in plant species.

### Functional annotation and comparative genomics

Functional annotations of GATA genes were enriched by comparing sequence homologies and conserved domains across plant species. Comparative genomics approaches were utilized to identify evolutionary conserved regions and variations specific to different types of GATA domains, such as IVb, IVc, IVa, and IV4.

## Supplementary Information

Supplementary Table S1.

Supplementary Table S2.

Supplementary Table S3.

Supplementary Table S4.

## Data Availability

All GATA TFs identified in this study can be accessed at http://planttfdb.gao-lab.org/.
