# Author Correction: Comparative analysis of amino acid sequence level in plant GATA transcription factors

**DOI:** 10.1038/s41598-025-93092-4

**Published:** 2025-03-14

**Authors:** Mangi Kim

**Affiliations:** Department of Biotechnology, Sangmyung University, 03016 Seoul, Republic of Korea

Correction to: *Scientific Reports* 10.1038/s41598-024-81159-7, published online 30 November 2024

The original version of this Article contained an error in Fig. 4, panel **(d)**, where the count of the GATA domain types (Type IVa, Type IVb, Type IVc, Type IVe, Type IV4, Type IVp) for the six Orders in Monocot and Basal Angiosperm was incorrect.

The original Fig. 4 and accompanying legend appear below.

**Fig. 4:**
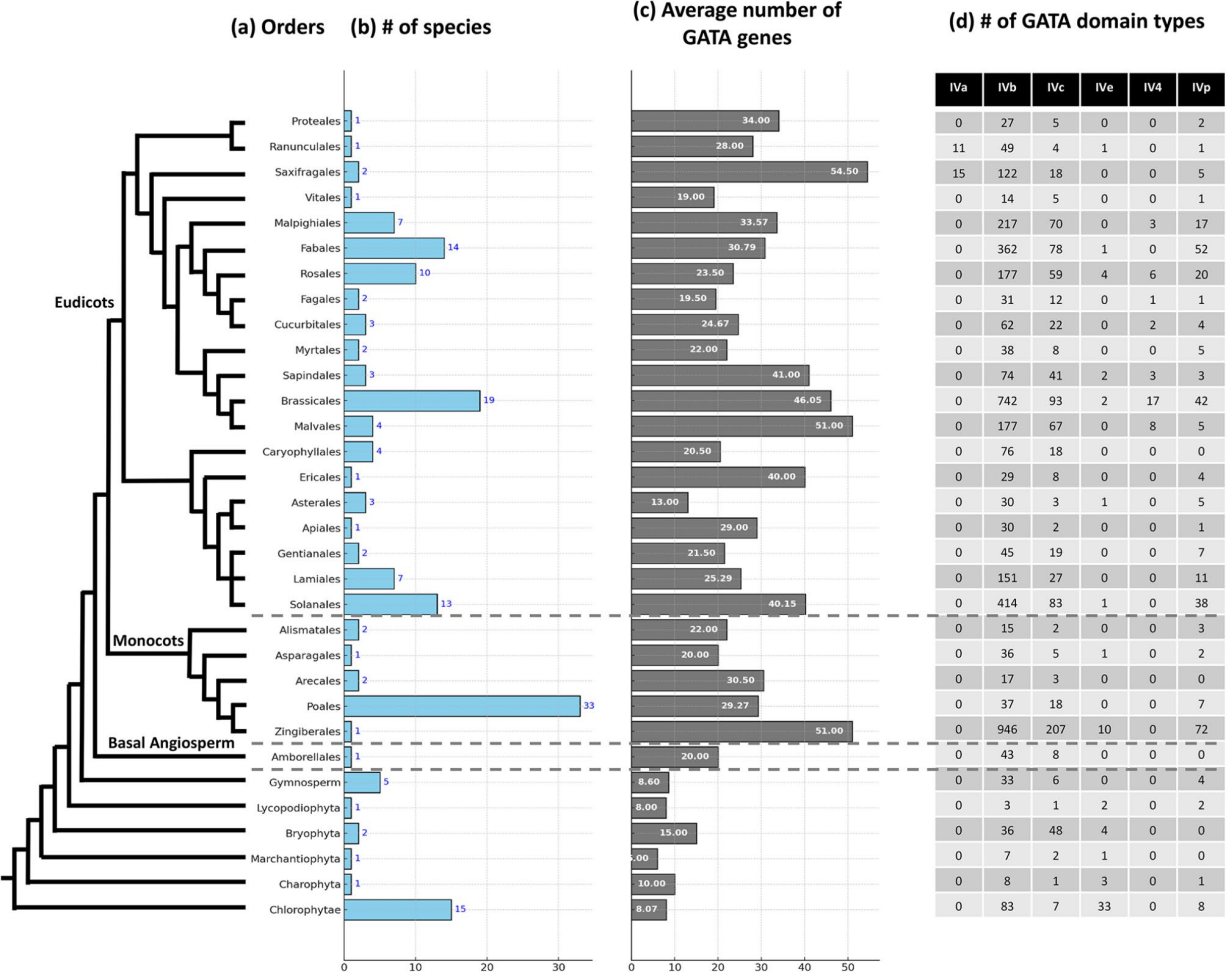
Phylogenetic distribution and characteristics of GATA genes and domain types across plant orders. The figure displays the distribution and characteristics of GATA genes and domain types across various plant orders. (**a**) The phylogenetic tree illustrates the evolutionary relationships among the major plant groups, including eudicots, monocots, basal angiosperms, and gymnosperms. (**b**) The bar graph shows the number of species sampled within each order. (**c**) The average number of GATA genes per species in each order is presented, with values annotated on the bars. (**d**) The table lists the distribution of GATA domain types (IVa, IVb, IVc, IVe, IV4, and IVp) for each order, providing insight into the diversity of GATA domains within and across groups.

The original Article has been corrected.

